# Modulation of plantar pressure and gastrocnemius activity during gait using electrical stimulation of the tibialis anterior in healthy adults

**DOI:** 10.1371/journal.pone.0195309

**Published:** 2018-05-10

**Authors:** Maiki Moriguchi, Noriaki Maeshige, Mizuki Ueno, Yoshiyuki Yoshikawa, Hiroto Terashi, Hidemi Fujino

**Affiliations:** 1 Department of Rehabilitation Science, Kobe University Graduate School of Health Sciences, Tomogaoka, Suma-Ku, Kobe, Hyogo, Japan; 2 Miyabinosato Home-visit nursing care station, Patio Akashi 1F, Uozumicho, Nakao, Akashi, Hyogo, Japan; 3 Department of Plastic Surgery, Kobe University Graduate School of Medicine, Kusunokicho, Chuo-Ku, Kobe, Hyogo, Japan; The Ohio State University, UNITED STATES

## Abstract

High plantar flexor moment during the stance phase is known to cause high plantar pressure under the forefoot; however, the effects on plantar pressure due to a change of gastrocnemius medialis (GM) activity during gait, have not been investigated to date. Reciprocal inhibition is one of the effects of electrical stimulation (ES), and is the automatic antagonist alpha motor neuron inhibition which is evoked by excitation of the agonist muscle. The aim of this study was to investigate the influences of ES of the tibialis anterior (TA) on plantar pressure and the GM activity during gait in healthy adults. ES was applied to the TAs of twenty healthy male adults for 30 minutes at the level of intensity that causes a full range of dorsiflexion in the ankle (frequency; 50 Hz, on-time; 10 sec, off-time; 10 sec). Subjects walked 10 meters before and after ES, and we measured the peak plantar pressure (PP), pressure time integral (PTI), and gait parameters by using an F-scan system. The percentage of integrated electromyogram (%IEMG), active time, onset time, peak time, and cessation time of TA and GM were calculated. PP and PTI under the forefoot, rear foot, and total plantar surface significantly decreased after the application of ES. Meanwhile, changes of gait parameters were not observed. %IEMG and the active time of both muscles did not change; however, onset time and peak time of GM became significantly delayed. ES application to the TA delayed the timing of onset and peak in the GM, and caused the decrease of plantar pressure during gait. The present results suggest that ES to the TA could become a new method for the control of plantar pressure via modulation of GM activity during gait.

## Introduction

High plantar pressure caused by foot deformities (e.g. hammer or claw toe, hallux valgus, bony prominences and/or Charcot arthropathy) and limited ankle dorsiflexion range of motion (ROM) are risk factors for the formation of callosities on the plantar surface [1,2]. In elderly individuals, callosities are a common cause of foot pain, which affects the activities of daily living (ADL) and increases the risk of falling [3]. Especially in patients with peripheral neuropathy, the lack of a protective sensation that results from this condition leads to unattended minor injuries caused by repetitive excess plantar pressure via callosities [4]. Once the skin has been breached, the healing process is impaired on a broken area by continued mobilization, which leads to the infection of superficial and deeper tissues [5], resulting often in lower limb amputation [6,7]. Therefore, the reduction of higher degree of plantar pressure is important for limb salvage and for maintaining quality of life.

Diabetes mellitus (DM) is a major disease that causes neuropathy in the feet. The presentation of patients with diabetic peripheral neuropathy (DPN) is characterized by an increase of plantar pressure caused by several structural alterations, autonomic, sensory, and motor deficits [8,9]. Various interventions for the reduction of high plantar pressure, such as certain footwear, insoles and control methods for gait speed have been reported and evaluated in clinical care settings; however, annual ulcer recurrence rates remain high, up to 40% [10]. Footwear is effective in the prevention of a recurrent foot ulcer [11], and the combination of a low peak pressure with footwear (< 200 kPa) and high adherence (> 80%) can reduce risk of ulcer recurrence by more than 50% [11]. Adherence is calculated as the percentage of steps wearing prescribed footwear over the cumulative number of steps during the full measurement periods. It has been reported that the patients with adherence rates < 80%, account for approximately 50% of the total subjects; and that low adherence of wearing prescribed intervention is major risk factor for recurrent lesions in patients with DM [12]. Therefore, novel strategies to reduce plantar pressure, while DM patients live without footwear, also need to be developed. Moreover, a new intervention combined with therapeutic footwear can relieve plantar pressure despite low adherence of footwear, resulting in the decrease of risk for recurrence.

The alterations of gait kinetics and kinematics caused by peripheral neuropathy contributes to changes in plantar pressure distribution [13]. Among these alterations, the increased plantar flexor moment during mid-stance is one of the changes that causes the high plantar pressure under the forefoot [9]. Plantar flexor moment during gait is produced by the activity of the triceps-surae muscle-tendon complex (TS-MTC) [14]. The gastrocnemius medialis (GM) is a muscle that composes the TS-MTC. Modeling studies suggest that the GM is a primary contributor to forward acceleration in walking, and that the medial and lateral gastrocnemius muscles combined contribute more to forward propulsive forces during stance than the soleus [15,16]. Early generation of propulsive force induces the early forward shift of the center of pressure and thus the increase of the duration under plantar loading in the forefoot. Therefore, a decrease in the activity of GM during mid-stance could suppress the plantar pressure under the forefoot.

Reciprocal inhibition is one of the effects of electrical stimulation (ES) therapy, and it reduces the activity of the antagonist muscle by agonist muscle stimulation [17]. Indeed, Koyama et al. reported that the H-reflex of the soleus significantly decreased after ES application to the tibialis anterior (TA) [18]. Thus, it can be argued that ES can affect directly the muscle activity; therefore, the application of ES to the TA prior to physical movement could facilitate reciprocal inhibition to GM during the following action of walking.

We hypothesized that the application of ES to the TA suppresses GM activity during the stance phase, and that this inhibition leads to the decrease of plantar pressure in the subsequent gait; however, this physiological change has not been reported to occur either in healthy subjects or in patients. Therefore, the present study is conducted to survey the influence of ES application to the TA on plantar pressure and the GM activity pattern in healthy adults as a preliminary study for applying ES to patients with DM.

## Methods

### Subjects

The subjects of this study were 20 healthy male adults (mean age 24.4 ± 5.7 years old; mean height 174.6 ± 5.2 cm; mean weight 66.1 ± 4.4 kg). All subjects reported no history of either neurological or orthopedic impairments. These experiments were approved by the Ethics Committee of the Kobe University, and all subjects signed an informed consent form. This study procedure was performed in accordance with the Declaration of Helsinki.

### Study procedure

All subjects were instructed to refrain from alcohol and strenuous activity for 24 hours prior to the experiments. ES was applied to the TA of the subjects for 30 minutes, while they were positioned sitting on the bed. First, we applied passive force until firm resistance to further movement was encountered, and then we measured ankle dorsiflexion ROM with knee full extension and 90° flexion using a hand-held goniometer. We measured the ankle dorsiflexion ROM, and subjects were directed to walk 10 m four times at a self-selected walking speed resulted within 1.45 ± 1.77 m/s (normal walking speed in young adults) [19] to measure plantar pressure and muscle activity during gait before and after ES [20].

### Electrical stimulation

Subjects' TAs were stimulated using an electrical stimulation device (ES-360, ITO CO., Tokyo, Japan) set at 50 Hz with pulse duration of 300 μsec [21,22]. Stimulating electrodes (PALS Platinum M size, each about 5 cm long by 5 cm wide; Axelgaard Manufacturing Co., Fallbrook, CA, USA) were placed proximally and distally over the motor point of the TA muscles. In addition, we measured the dorsiflexion ROM per five minutes during ES, and regulated the stimulus intensity to keep the angle within -5° from the maximum dorsiflexion ROM.

### Plantar pressure measurement

Plantar pressure distribution was recorded using an F-scan in-shoe pressure measurement system (NITTA Co., Osaka, Japan) at 50 Hz during gait. We fixed sensor seats on the subjects’ plantar surfaces using extensibility tape and socks to minimize the slip of sensor. Calibration was performed using the patient's body weight, and with one leg standing on the sensor. According to the method of Sartor et al., the plantar surface was divided into three areas: the rear foot, midfoot, and forefoot [22]. Peak pressure (PP) and pressure time integral (PTI) were calculated for four areas (rear foot, midfoot, forefoot and total plantar surface).

Each data set was calculated per one gait cycle, and the average of four trials was taken to minimize subject variability. The interclass correlation coefficients in PP and PTI measurements were 0.95 and 0.98, respectively, indicating excellent reliability for the measurement of plantar pressure using the F-scan system.

### Gait parameters

Walking speed, stance time, swing time, and stride length were recorded using the F-scan as gait parameters during 10 m of walking. We identified the heel-strike (HS) as the moment at which any sensor in the rear foot area reacted. The foot-flat (FF) was identified as the duration from the moment at which any sensor in the forefoot area detected pressure to the moment all sensors that were detecting pressure in rear foot area ceased to. The toe-off (TO) was identified as the moment at which all sensors detecting pressure in the forefoot area ceased doing so. Based on these categorizations, we recorded the duration of each gait cycle as being from HS to the beginning of FF (HS-FF), during FF (FF), and from the end of FF to TO (FF-TO). Moreover, we calculated the sum of HS-FF and FF as the contact duration of rear foot, and the sum of FF and FF-TO as the contact duration of forefoot.

These data were calculated using the average of four trials in a manner similar to that used with plantar pressure. The ICCs of these measurements ranged from 0.88 to 0.98, indicating excellent reliability for the measurement of these gait parameters using the F-scan system.

### Electromyography

Electromyography (EMG) activities were recorded from the right GM and TA during gait using a two-channel data logger (Feedback Logger, DKH, Tokyo, Japan) and surface EMG sensor (SX230-1000, Biometrics Ltd, Newport., UK) at 1,000 Hz. We recorded GM activity because it was more sensitive than lateralis during gait in the preliminary study. The surface EMG sensors were placed according to the method that the SENIAM (Surface ElectroMyoGraphy for the Non-Invasive Assessment of Muscles) project [23] recommends. The load switch system was attached on the heel as a foot switch for determining HS, and the signals from the load switch system and F-scan system at HS had to be synchronized between plantar pressure data and EMG data.

Based on the method of Nagai et al., the EMG activities from GM and TA during maximum isometric contraction were recorded via maximum isometric dorsiflexion of the ankle with the subject sitting on the chair and maximum isometric plantar flexion with the patient in single leg stance, respectively. From these, we calculated the integrated electromyogram (IEMG) [24]. Root mean square (RMS) using a 50-msec window was calculated during stance phase of the gait cycle. Muscle activity onset, duration and cessation were determined using the threshold of 10% of maximum amplitude per gait cycle, which can detect the small activity of muscles during gait, excluding the artifact of baseline. Active time, onset time, peak time and cessation time were determined based on RMS wave. IEMG was shown as a percentage of the IEMG at maximum isometric contraction (%IEMG). Onset time, peak time, active time, and cessation time were reported separately as a percentage of the stance time.

EMG data obtained during gait were calculated based on the average of four trials, in a manner similar to that of plantar pressure.

### Statistical analysis

After the confirmation of normality and homoscedasticity of all data, parametric data were compared using paired t-test for comparison between before and after ES. Non-parametric data were compared using Wilcoxon signed-rank test. All analyses were performed with R version 3.2.5 (R Core Team (2014). R: A language and environment for statistical computing. R Foundation for Statistical Computing, Vienna, Austria, http://www.R-project.org/). The level of significance was set at *P* < 0.05.

## Results

### Plantar pressure

**Fig 1:**
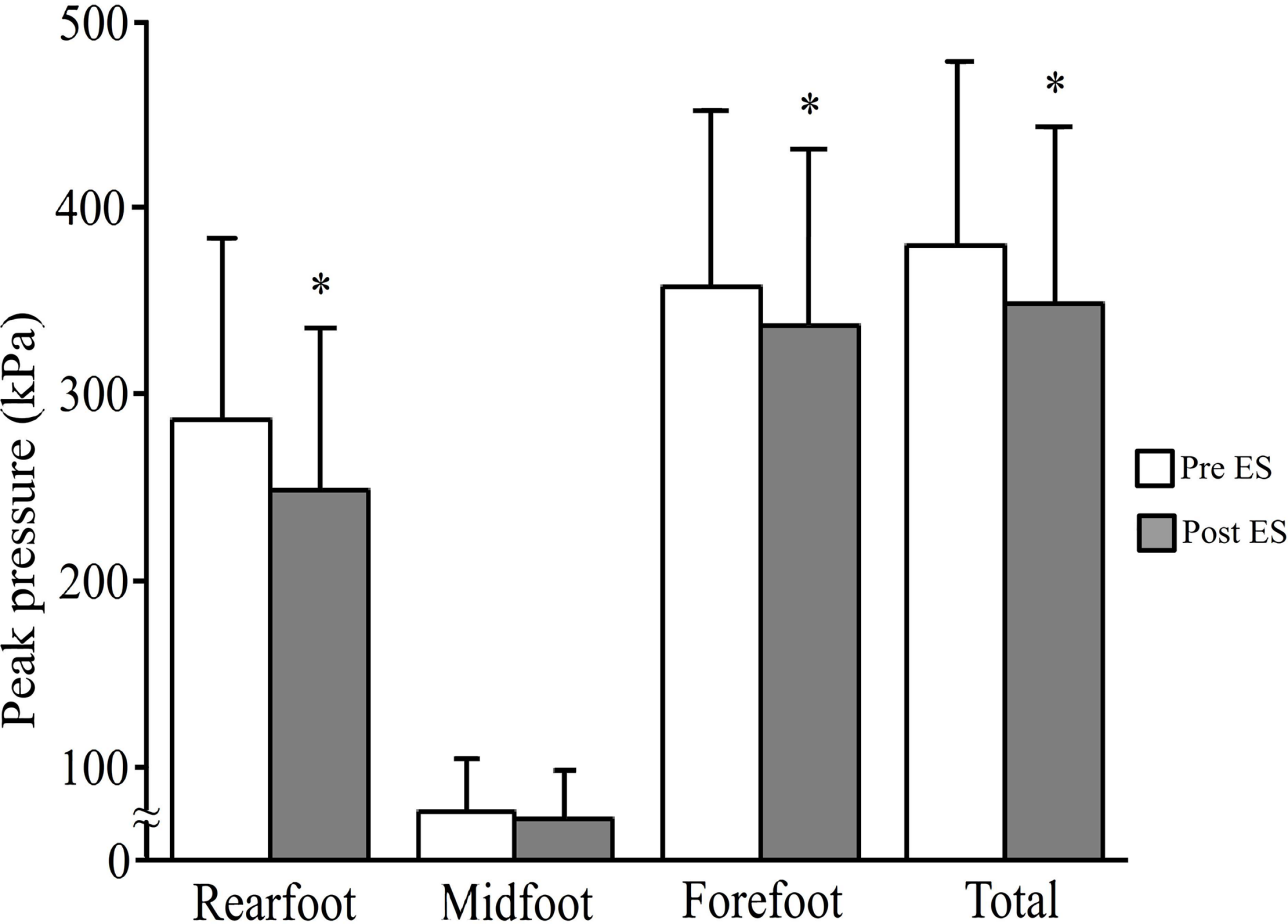
Peak pressure (PP) under the three regions (rear foot, midfoot, forefoot) and total plantar surface before and after ES application. The white bar indicates PP before ES application and the gray bar indicates PP after ES application. * represents a significant difference from pre-ES period (P < 0.05).

**Fig 2:**
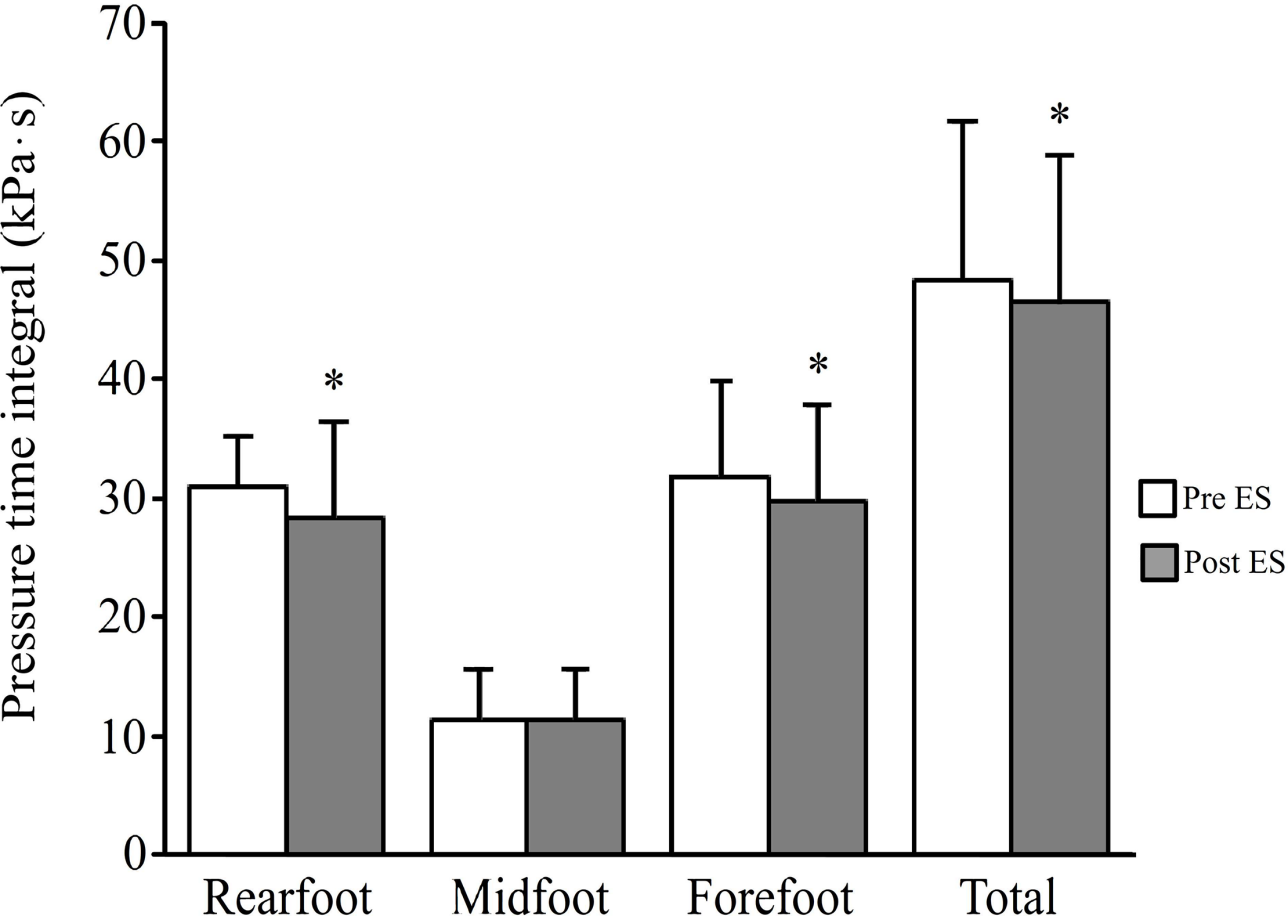
Pressure time integral (PTI) under the three regions (rear foot, midfoot, forefoot) and total plantar surface before and after ES application. The white bar indicates PTI before ES application and the gray bar indicates PTI after ES application. * represents a significant difference from pre-ES period (P < 0.05).

After ES application, PP (Fig 1) significantly decreased under the forefoot (from 357.5 ± 95.1 kPa to 336.9 ± 93.9 kPa; P < 0.05), rear foot (from 286.3 ± 97.2 kPa to 249.3 ± 86.2 kPa; P < 0.05) and total plantar surfaces (from 379.5 ± 99.6 kPa to 349.5 ± 93.9 kPa; P < 0.05). PTI (Fig 2) also significantly decreased under the forefoot (from 31.7 ± 8.1 kPa·s to 29.7 ± 8.1 kPa·s; P < 0.05), rear foot (from 31.0 ± 4.2 kPa·s to 28.3 ± 8.1 kPa·s; P < 0.05) and total plantar surfaces (from 48.4 ± 13.3 kPa·s to 46.6 ± 12.2 kPa·s; P < 0.05); however, PP (Fig 1) and PTI (Fig 2) under the midfoot did not change (PP: from 76.4 ± 28.1 kPa to 72.9 ± 25.3 kPa, PTI: from 11.5 ± 4.2 kPa·s to 11.3 ± 4.2 kPa·s).

### Ankle DF ROM and gait parameters

Ankle DF ROM did not change after the application of ES (Table 1).

**Table 1:** Dorsiflexion range of motion of ankle.

	pre ES			post ES			*P* value
Knee extension (°)	21.8	±	4.6	21.9	±	4.5	0.49
Knee 90° flexion (°)	32.2	±	6.3	32.2	±	6.2	0.68

Dorsiflexion range of motion were measured by manual goniometry.

Values are presented as mean ± standard deviation.

The walking speed, stride length, and other gait parameters also did not change significantly following the application of ES (Table 2).

**Table 2:** Spatiotemporal parameters during gait.

	pre ES			post ES			*P* value
Walking speed (m/s)	1.42	±	0.17	1.42	±	0.18	0.91
Number of steps (steps)	13.6	±	1.00	13.6	±	1.00	0.99
Stride length (m)	1.49	±	0.11	1.48	±	0.13	0.67
Time of stance-phase (s)	0.59	±	0.04	0.59	±	0.04	0.21
Time of swing-phase (s)	0.47	±	0.02	0.48	±	0.02	0.07

Values are presented as mean ± standard deviation.

**Fig 3:**
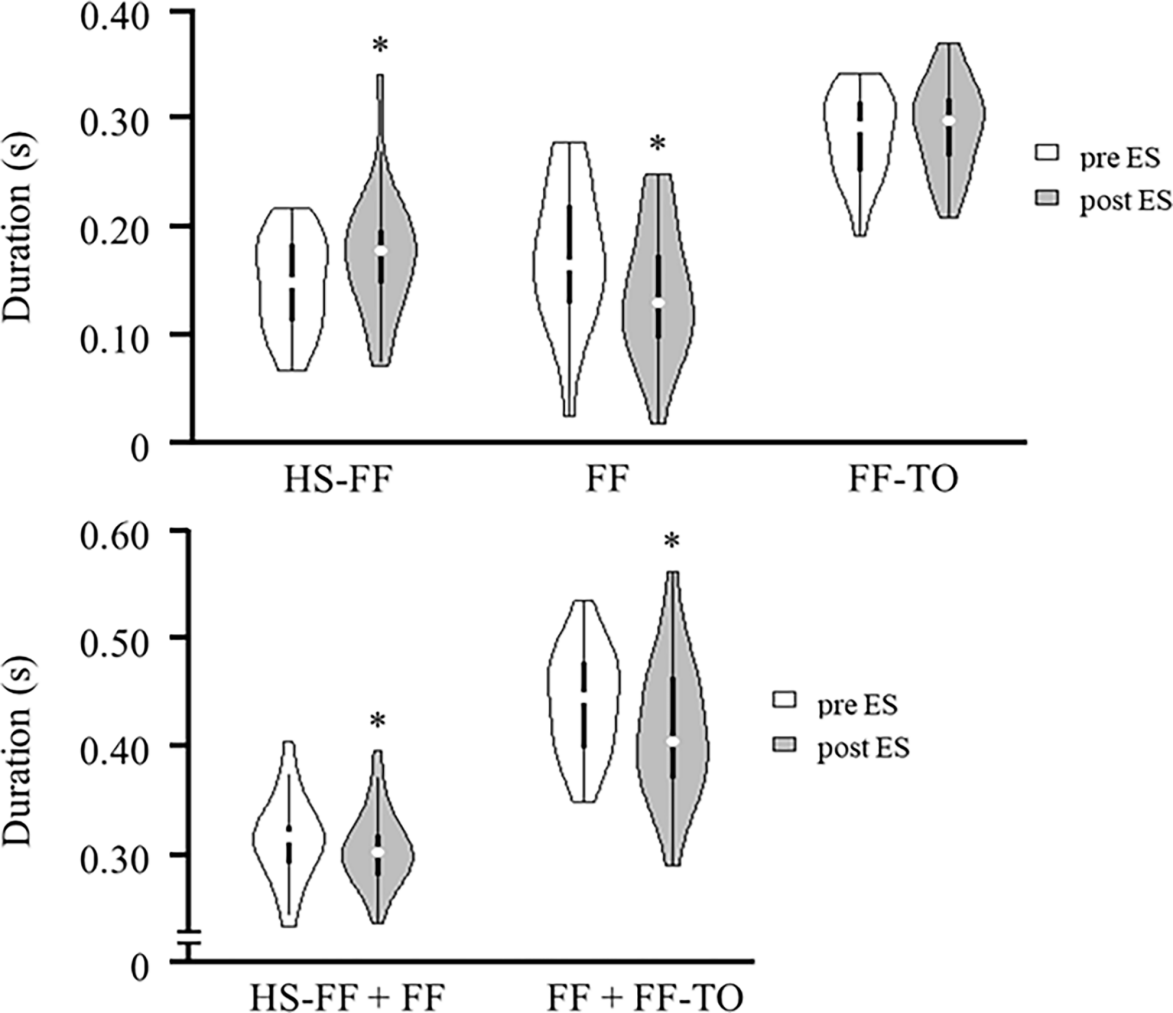
Duration of each gait cycle before and after ES application. The white one indicates HS-FF, FF, FF-TO, HS-FF + FF and FF + FF-TO before ES application. The gray one indicates these parameters after ES application. Density plot shows the distribution shape of the data. The thick black bar in the center represents the interquartile range, the thin black line extended from it represents the 95% confidence intervals, and the white dot is the median. The sum of HS–FF and FF is the contact duration of rear foot, and the sum of FF and FF–TO is the contact duration of forefoot. * represents a significant difference from pre-ES period (*P <* 0.05). Abbreviations: HS, heel strike; FF, foot-flat; TO, toe-off.

However, the duration of HS-FF significantly increased and FF significantly decreased following use of ES. In addition, the duration of FF + FF-TO, which means the contact duration of the forefoot during the stance phase, significantly decreased after ES use (Fig 3, S1 Table).

### Muscle activity

In all gait trials, TA was activated prior to HS, and the activity of GM started during TA activation. The cessation of TA activity was obtained at around 30% of the stance phase, and then TA was activated again at around 60% of the stance phase. The sequence of these activities was similarly obtained before and after the application of ES. Meanwhile, a significant delay of onset time and peak time of GM was obtained after ES use (Table 3). %IEMG, active time and cessation time of all muscle did not change after ES application (Table 3), and onset time and peak time of TA also did not change (Table 3)

**Table 3:** Normalized EMG data as activity of GM and TA during gait.

	GM						
	pre ES			post ES			*P* value
%IEMG (%)	7.0 (3.1–42.5)			8.4 (3.2–18.3)			0.91
Active time (%)	66.6	±	3.6	67.4	±	2.8	0.64
Onset time (%)	13.9	±	3.1	17.1	±	3.8*	0.03
Peak time (%)	49.6	±	3.4	58.1	±	1.6*	0.04
Cessation time (%)	83.7	±	1.4	84.8	±	0.8	0.47
	TA						
	pre ES			post ES			*P* value
%IEMG (%)	2.1 (1.1–48.9)			2.1 (1.1–51.1)			1
Active time (%)	60.4 (23.4–86.4)			67.6 (20.7–82.2)			0.25
Onset time (%)	65.5	±	6.7	68.5	±	5.6	0.38
Peak time (%)	3.3	±	0.5	3.2	±	0.6	0.59
Cessation time (%)	28.2	±	5.4	25.7	±	5.3	0.18

Abbreviations: GM, gastrocnemius medialis; TA, tibialis anterior: ES, electrical stimulation; IEMG, integrated electromyogram.

Values are presented as mean ± standard deviation, or median, in parenthesis.

* represents a significant difference from pre-ES period (*P <* 0.05).

## Discussion

In the present study, we found that the regulation of GM activity occurred by application of ES to the TA, in addition to the appearance of a suppressive effect on plantar pressure during gait, in healthy male adults. This is the first report about such plantar pressure regulation by ES, and this suggests that the application of ES to the TA could be an effective method for plantar pressure suppression and a therapeutic intervention for several disorders caused by the presence of high plantar pressure during gait.

PP not only under the forefoot but also under the rear foot and total plantar surface decreased after ES use in the present study. Although we hypothesized that the reduction of GM activity via reciprocal inhibition decreases plantar pressure under the forefoot, plantar pressure decreased without the reduction of GM activity, as demonstrated in %IEMG in the present study. Meanwhile, we identified the delay of onset and peak time of GM activity after ES. Kwon et al. reported that the premature activation of GM was one of the contributing factors to increase impulse at the forefoot [25]. Therefore, the delay in GM activity would contribute to the pressure decrease under the forefoot in the present study. Honeine et al. reported that triceps surae activity burst at approximately 60% of the stance phase contributed to a reduction in the downward center of mass (CoM) [26]. In the present study, the peak time of GM was delayed from 49.6 ± 3.4% to 58.1 ± 1.6% by the application of ES. Therefore, it is speculated that this delay in peak time of GM led to the efficient action of GM to suppress the downward shift of CoM. CoM excursion during gait is calculated based on the integral of ground reaction force (GRF) with respect to time, and strong correlation (R = 0.84) between vertical CoM excursion calculated using GRF and using segmental kinematic centroid model was reported [27]. In addition, the very strong correlation (R = 0.99) has been reported between the vertical GRF calculated using plantar pressure and using force plate [28]. Therefore, the alteration of the sum of plantar pressure under the total plantar surface reflects the change of vertical CoM excursion. Interestingly, PTI under the total plantar surface decreased after the application of ES in the present study. Hence, it is suggested that the delay in peak time of GM suppresses the downward shift of CoM, resulting in decreases of PP under the forefoot, rearfoot, and total foot surface”.

In the present study, ES application decreased the PTIs under the forefoot and the rear foot. PTI changes depend on alterations in pressure magnitude and contact duration because PTI is the sum of the pressure value per unit time. In the present study, ES application decreased PPs under the forefoot and the rear foot, as well as the duration of contact in each lesion. Therefore, PTIs under the forefoot and the rear foot were decreased by the reduction of both pressure value and contact duration. Interestingly, we found an increase in the duration of HS-FF after ES in the present study. As for this alteration, we focused on the moment of plantar flexion. This moment is generated at HS by the vertical GRF applied to the rear foot; that is, the reduction of vertical GRF at HS decreases the moment of plantar flexion. In the present study, the decrease of PP under the rear foot led to the decrease of plantar flexion moment at HS, resulting in the increase of the duration of HS-FF. PTI under the total plantar surface also decreased in the present study. This change is due to the PTI decrease in forefoot and rear foot.

Onset time and peak time of GM was significantly delayed after ES application in the present study, suggesting that ES to TA changed the GM activity pattern during gait. Koyama et al. reported that the amplitude of the H-reflex of TS during and after application of ES to the TA was significantly lower than that seen prior to ES use [18]. The H-reflex reflects the monosynaptic Ia excitation of motor neurons [18]; therefore, the decrease of the amplitude of the H-reflex means the reduction of α-motor neuron excitation, resulting in the inhibition of stretch reflex. Chalmers et al. reported the contribution of the stretch reflex by the ankle extensor muscle stretch in the first half of stance phase to plantar flexor force production [29]. Therefore, the application of ES to the TA would delay GM activity via stretch reflex inhibition in the early stance phase induced by reciprocal inhibition, as in the present study.

Finally, plantar pressure decreased after ES without any change of gait parameters in this study. Some studies reported that walking speed and stride length have a proportional relation to plantar pressure [30,31]. Furthermore, Robinson et al. reported decreases in PP as well as walking speed in patients with DM [32]. In contrast with these studies, plantar pressure decreased without any changes in gait parameters in our study. According to the study that investigated the contribution of joint moment in the lower limbs to walking speed, the moment peak of the hip joint showed a strong correlation with walking speed as compared with other joint moments. Meanwhile, the correlation between ankle joint moment and walking speed was found to be weak after mid-stance [33]. Therefore, the alteration in ankle muscle activity would not necessarily affect gait parameters.

The present study involving healthy adults was conducted to survey the physiological effect of ES application on plantar pressure during gait, and it is uncertain what the effect of ES in the patients with DM would be. Furthermore, the persistence of beneficial effects from ES is unknown, it is difficult to say if they can prevent increased plantar pressure during normal daily activity. Koyama et al. found that the decrease of the amplitude of H-reflex continued until 15 minutes after ES application [18], and hence ES may decrease plantar pressure for 15 minutes after the use of ES. However, ES of muscle has been shown to enhance motor learning, and neuronal activity in sensorimotor regions of the brain [34]. Therefore, patients with DM may acquire gait patterns preventing the increase of plantar pressure by long-term intervention of ES.

In future, the acute effect of ES application, the duration of this ES effect, and the acquisition of gait pattern in patients with DM needs to be investigated for the development of the strategy for the clinical treatment.

## Supporting information

S1 Table Duration of each gait cycle before and after ES application. Abbreviations: CI, confidence interval; HS, heel strike; FF, foot-flat; TO, toe-off.
Values are presented as mean (standard deviation), median (interquartile range).
* represents a significant difference from pre-ES period (*P <* 0.05).
The sum of HS–FF and FF is the contact duration of rearfoot, and the sum of FF and FF–TO is the contact duration of forefoot.
(DOCX)

S1 Dataset The duration of each gait cycle. (XLSX)
