# Supplementary material for: Modulation of plantar pressure and gastrocnemius activity during gait using electrical stimulation of the tibialis anterior in healthy adults
Source: PLoS One. 2018 May 10;13(5):e0195309. doi: 10.1371/journal.pone.0195309 (PMC5944963; doi:10.1371/journal.pone.0195309)
Supplement: S1 Table — Abbreviations: CI, confidence interval; HS, heel strike; FF, foot-flat; TO, toe-off. Values are presented as mean (standard deviation), median (interquartile range). * represents a significant difference from pre-ES period (P < 0.05). The sum of HS–FF and FF is the contact duration of rearfoot, and the sum of FF and FF–TO is the contact duration of forefoot. (DOCX) [file pone.0195309.s001.docx]

**Table supplement 1.** Duration of each gait cycle before and after ES application

|  | Pre-ES | | | | Post-ES | | | |
| --- | --- | --- | --- | --- | --- | --- | --- | --- |
|  | Mean  (SD) | Median  (interquartile) | Range  (min - max) | 95% CI  (lower - upper) | Mean  (SD) | Median  (interquartile) | Range  (max - min) | 95% CI  (lower- upper) |
| HS–FF(s) | 0.15  (0.04) | 0.15  (0.11 – 0.18) | 0.07 – 0.22 | 0.13 – 0.17 | 0.17*  (0.06) | 0.18  (0.15 – 0.20) | 0.07 – 0.34 | 0.15 – 0.20 |
| FF (s) | 0.17  (0.07) | 0.16  (0.12 – 0.21) | 0.02 – 0.27 | 0.14 – 0.20 | 0.13*  (0.06) | 0.12  (0.09 – 0.17) | 0.01 – 0.24 | 0.10 – 0.16 |
| FF–TO (s) | 0.28  (0.04) | 0.28  (0.24 – 0.31) | 0.18 – 0.33 | 0.26 – 0.29 | 0.28  (0.04) | 0.29  (0.26 – 0.31) | 0.20 – 0.36 | 0.26 – 0.30 |
| HS–FF + FF (s) | 0.31  (0.04) | 0.31  (0.29 – 0.32) | 0.23 – 0.40 | 0.29 – 0.33 | 0.30*  (0.04) | 0.30  (0.28 – 0.32) | 0.23 – 0.40 | 0.29 – 0.32 |
| FF + FF–TO (s) | 0.44  (0.05) | 0.45  (0.40 – 0.48) | 0.35 – 0.54 | 0.42 – 0.46 | 0.42*  (0.06) | 0.41  (0.37- 0.46) | 0.29 – 0.56 | 0.39 – 0.45 |

Abbreviations: CI, confidence interval; HS, heel strike; FF, foot-flat; TO, toe-off.

Values are presented as mean (standard deviation), median (interquartile range).

* represents a significant difference from pre-ES period (*P <* 0.05).

The sum of HS–FF and FF is the contact duration of rearfoot, and the sum of FF and FF–TO is the contact duration of forefoot.
